# Design and Optimization of Polyaniline/SWCNT Anodes for Improved Lithium-Ion Storage

**DOI:** 10.3390/polym17040478

**Published:** 2025-02-12

**Authors:** Keshavananda Prabhu Channabasavana Hundi Puttaningaiah

**Affiliations:** Department of Chemical, Biological and Battery Engineering, Gachon University, Seongnam-si 13120, Gyeonggi-do, Republic of Korea; keshavmgm@gmail.com

**Keywords:** polyaniline (PANI), single-walled carbon nanotubes (SWCNTs), lithium-ion batteries (LIBs), composite materials, electrochemical performance, energy storage, cyclic voltammetry (CV), charge–discharge performance

## Abstract

The integration of polyaniline (PANI) with single-walled carbon nanotubes (SWCNTs) offers a promising technique to improve the electrochemical performance of lithium-ion battery (LIB) anodes. In this work, we report on the synthesis and advanced optimization of PANI/SWCNT composite anodes aimed toward further developing lithium-ion (Li^+^) storage capacity. A proper characterization, including SEM and XRD, revealed the well-defined morphology and synergistic collaboration among PANI and SWCNTs. Electrochemical evaluations showed that the PANI anodes display predominant Li^+^ storage capacities, with a high specific capacity of 528 mA g^−1^ at 100 mA g^−1^, and the 10 wt% SWCNT-doped PANI (PANI/10 wt% SWCNT) composite demonstrated an exceptional cycling performance of 830 mA g^−1^ at 100 mA g^−1^ and excellent capacity retention (101% after 200 cycles). Cyclic voltammetry demonstrated reduced charge transfer resistance and improved ion diffusion kinetics. These improvements originate from the correlative properties of PANI’s redox activity and SWCNT’s conductivity, which enable effective Li^+^ transport and intercalation. This work features the capability of PANI/SWCNT composites as superior-performance anode materials for advanced LIBs, tending to key difficulties of energy density and cycling stability. The discoveries establish the importance of additional investigation of polymer–carbon nanocomposites in advanced energy storage systems.

## 1. Introduction

The developing demand for energy storage solutions to power portable electronics, electric vehicles, and grid applications has spurred much exploration into lithium-ion batteries (LIBs) [1,2,3,4,5]. Despite their commercial achievement, regular LIBs face constraints and limitations in energy density, cycle life, and safety, especially according to the performance of their electrode materials [6,7,8]. Developing high-capacity, stable, and efficient anodes is critical for advancing advanced LIB innovation technology [9]. In this unique situation, composite materials that consolidate polymers and nanostructures offer a promising pathway to improving battery performance [10,11,12,13]. PANI, a conductive polymer, has garnered consideration because of its excellent electrical conductivity, environmental stability, and intrinsic redox activity [14,15]. These properties empower PANI to participate in charge storage applications, potentially improving the specific capacity of LIB anodes [16,17,18,19,20]. However, PANI’s practical application is ruined by difficulties such as low cycling stability and poor mechanical durability, particularly under repeated lithiation/de-lithiation cycles [19]. SWCNTs, described by their high electrical conductivity, large surface area, and mechanical strength, act as optimal and ideal partners to PANI in composite materials [20,21,22,23]. SWCNTs provide an interconnected conductive organization that facilitates fast electron transport while relieving the volume changes associated with PANI during cycling [22]. The synergistic combination of PANI and SWCNTs in a composite grid can address individual material shortcomings, bringing about anodes with further developed Li^+^ storage and cycling performance.

PANI/SWCNT composite exhibited remarkable physical and compound properties because of the synergistic combination of PANI’s redox activity and SWCNTs’ high conductivity and mechanical strength. Physically, the composite highlights upgraded electrical conductivity, expanded mechanical power, and a high surface area with porosity, working with charge transport and electrolyte diffusion. Chemically, it shows excellent redox activity with stable charge storage, improved electrochemical stability because of SWCNT support, and areas of strength for and cooperation with π-π stacking and hydrogen bonding. These properties make PANI/SWCNT composites highly suitable for energy storage devices, electrochemical sensors, and catalytic applications, where high conductivity, stability, and surface functionality are crucial.

This study focuses on the design, synthesis, optimization, and enhancement of PANI/SWCNT composites for LIB anodes. By utilizing advanced in situ polymerization strategies and material engineering, we aim to expand the electrochemical collaboration among PANI and SWCNTs. Exhaustive portrayal procedures are utilized to clarify the primary structure and morphological properties of the composites, while electrochemical examinations survey their performance under different cycling conditions. The discoveries of this work are expected to add to the improvement of elite performance anodes for LIBs, offering a sustainable approach to addressing the tendencies, challenges, and difficulties of energy density and durability in energy storage frameworks. The optimization procedures and strategies introduced here serve as an outline for utilizing polymer-nanostructure interactions in next-generation advanced battery materials.

## 2. Materials and Methods

### 2.1. Synthesis of PANI

The PANI/SWCNT composite synthesis includes a few key materials, each playing a basic and critical role in ensuring the successful development of the composite and its improved electrochemical performance. Initially, 0.2 M aniline monomer was mixed with 1 N hydrochloric acid. This aniline serves as the precursor for PANI, a conductive polymer, which frames the active material in the composite. A solution of 0.25 M of ammonium persulfate (APS) was added dropwise into the mixture. The APS was utilized as the oxidizing agent that works with the polymerization of aniline into PANI, ensuring the development of a conductive polymer organization. The whole mixture was allowed to continuously stir for 8 h at room temperature. The precipitate formed and separated out by filtration and was washed with distilled water and acetone. The obtained final product was dried at 60 °C for 24 h. The final product was ground completely, and the resultant product was used as an anode material for LIB and the yield was 86%.

### 2.2. Preparation of PANI, PANI/SWCNT Composite Electrode Sheets and Assembly of Battery Cells

The SWCNT was purchased from Nano Solution Company (SA210) with a diameter of 1.4~1.7 nm and a purity of >95 vol%. The mixture was prepared by combining synthesized PANI, Carbon dark (SP), and poly (acrylic acid) (PAA) binder in a 70:15:15 (*w*/*w*/*w*) ratio. Similarly, for the preparation of PANI/SWCNT, a composite electrode synthesized PANI, SWCNT, Carbon dark (SP), and poly (acrylic acid) (PAA) binder in a 70:10:5:15 (*w*/*w*/*w*) ratio, with ethanol added as a solvent. The resultant slurry was applied to a copper sheet and left to dry for 24 h. Circular pieces measuring 1.23 cm in diameter were then cut from the coated sheet and used as a working electrode. To assemble the LIB, lithium metal (99.9%, metal basis, 0.75 mm thick; Alfa Aesar, Republic of Korea) was used as the counter-electrode, while 1 M of LipF_6_ dissolved in 1:1 (*v*/*v*) mixture of ethylene carbonate (EC) and diethyl carbonate (DEC) served as the electrolyte. A polyethylene was employed as a separator. The battery was constructed as a coin-type cell (CR-2032) was assembled in a glove box to avoid exposure to oxygen and moisture.

### 2.3. Characterization

Structural characterization and Morphological studies of PANI and PANI/SWCNT composite were carried out using advanced imaging and diffraction procedures. Scanning Electron Microscopy (SEM) was used to investigate the scattering of SWCNTs inside the composite and the consistency of the PANI coating. These methodologies gave significant data on surface morphology and confirmed that the PANI polymer and SWCNTs were uniformly coated on the electrode surface, making a well-integrated, incorporated, and facilitated structure that is basic for enhancing electrochemical performance. Additionally, X-beam diffraction (XRD) was performed to inspect the crystalline design of the composite. XRD analysis revealed the successful integration of the two components without compromising their primary structural integrity. Electrochemical performance was examined using several methods to assess the practicality and viability of the PANI/SWCNT composite as an anode material for LIBs. Cyclic Voltammetry (CV) was used to focus on the redox conduct of the composite, revealing the efficient Li^+^ intercalation and de-intercalation processes worked with by the composite’s design. Electrochemical Impedance Spectroscopy (EIS) was coordinated to investigate the charge transfer resistance and particle dispersion inside the composite. The results showed lower impedance values diverged from PANI, highlighting further developed conductivity and quicker particle transport facilitated by the SWCNTs. Finally, charge–discharge tests were performed to assess the high specific capacity limit and cycling stability of the composite. The tests showed that the composite had a high specific capacity and great cycling stability, further exhibiting its potential as a high-performance anode material for LIBs.

## 3. Results and Discussion

### 3.1. Morphology and Structural Analysis

**Figure 1:**
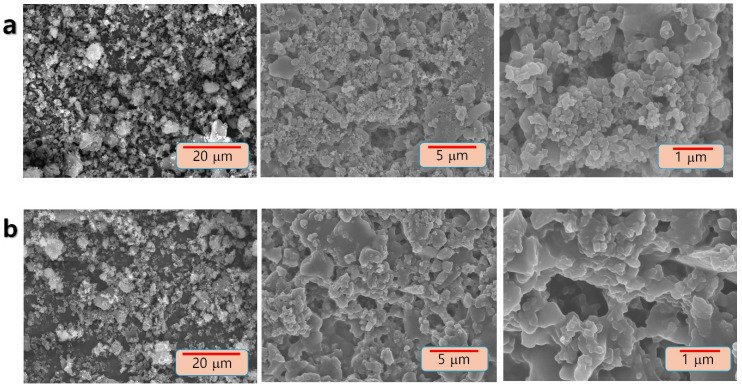
SEM image of (**a**) PANI and (**b**) PANI/SWCNT.

The primary and morphological assessments of the PANI/SWCNT composite gave critical insights into the high-level plan of the material. SEM pictures (Figure 1) uncovered a homogeneous and uniform covering of PANI on SWCNTs, outlining a strong conductive systems administration association. This covering is basic for guaranteeing productive electron and particle transport inside the composite, as it upgrades the material’s general conductivity and electrochemical activity and keeps up with effective Li^+^ transport pathways, which are fundamental for working on the by and large electrochemical execution of the anode material.

**Figure 2:**
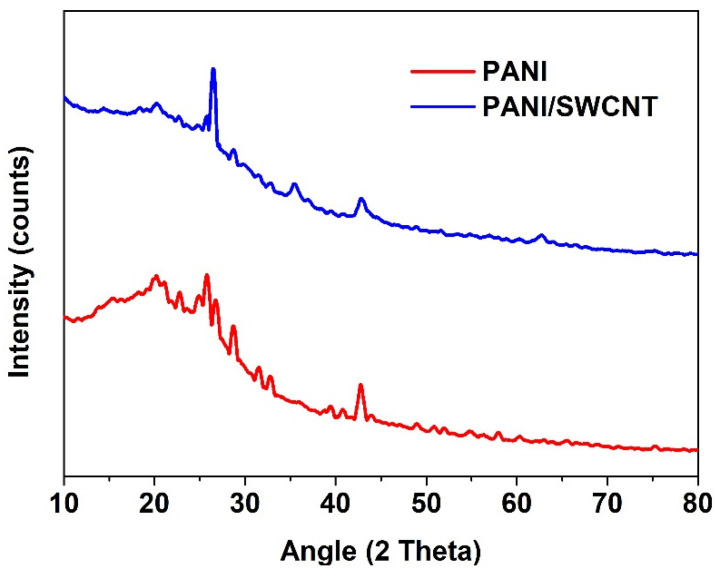
XRD pattern for the PANI and PANI/SWCNT.

XRD analyses were recorded in a 2θ-angle range of 5°–90° for both PANI and PANI/SWCNT, as displayed in Figure 2. The subsequent diffractogram gave further confirmation of the effective arrangement of the composite material. The XRD designs showed that the characteristic noise patterns proposed by the amorphous nature of the synthesized and composite materials. The amorphous properties of PANI and its composites are the key discoveries. These structural features play a crucial role in deciding the materials’ properties and potential applications in different fields, for example, gas sensors, energy materials, and electrical conductivity [22,23,24,25]. Together, the SEM and XRD results feature the effective incorporation of PANI and SWCNTs into a well-structured composite with optimized morphology for Li^+^ storage applications.

### 3.2. Electrochemical Performance

**Figure 3:**
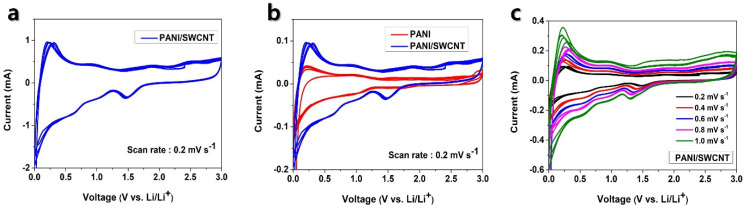
(**a**) Initial four cycle of the cyclic voltammetry curve for PANI/SWCNT at 0.2 mV s^−1^, (**b**) First four cycle of CV curves comparing PANI and PANI/SWCNT composite at 0.2 mV s^−1^, (**c**) CV curve of PANI/SWCNT electrode at different scans (0.2 to 1.0 mV s^−1^).

The electrochemical performance of the PANI/SWCNT composite was evaluated through several methods, featuring its enhanced functionality for LIB applications. In the cyclic voltammetry examination, the composite showed that four major redox peaks at 0.21/0.25 V, 0.94/0.63 V, 1.81/1.47 V, and 2.47/2.83 V (Figure 3a) were conducted at 0.2 mV s^−1^ with a voltage range of 0.01 to 3.0 V. The CV curves for PANI and PANI/SWCNT at 0.2 mV s^−1^ are illustrated in Figure 3b. The PANI/SWCNT has distinct and sharp redox peaks, which demonstrated effective Li^+^ intercalation and de-intercalation. These peaks were remarkably more intense compared with the individual PANI electrodes, suggesting that the PANI/SWCNT composite construction facilitated superior redox activity. This improved and enhanced redox performance is attributed to the synergistic interaction between PANI’s conductive backbone and the high surface area of SWCNTs, both of which support more proficient and efficient Li^+^ storage and delivery. As shown in Figure 3c, the CV shape remained consistent across various scan rates, indicating that the electrochemical energy storage capacity was improved due to the capacitance effect.

**Figure 4:**
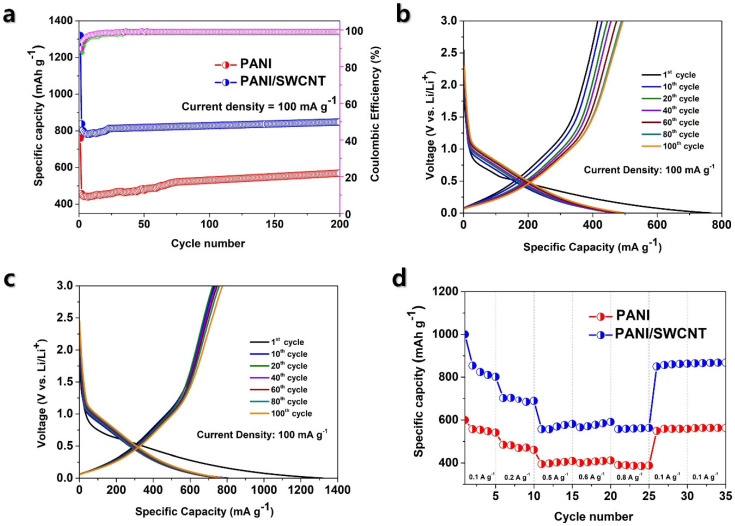
(**a**) Cycle performance comparison of PANI and PANI/SWCNT, (**b**) PANI galvanostatic charge–discharge (GCD) measurement profile at various cycles, (**c**) PANI/SWCNT GCD profile at different cycles, (**d**). Rate capacity of PANI and PANI/SWCNT electrodes under varying current densities.

Figure 4a shows a cycling performance of PANI and PANI/SWCNT at 100 mA g^−1^. This analysis exhibited impressive results, with the composite achieving a high specific capacity of 830 mA g^−1^ at 100 mA g^−1^, which is higher than the specific capacity of pure PANI (red line) and SWCNT(blue line) electrodes. Green and purple line indicates the Coulombic efficiency of the PANI and PANI/SWCNT electrode respectively. Table S1 exhibits the comparative cycle performance of initial discharge capacity, first discharge capacity, and capacity retention after 100 cycles for PANI and PANI/SWCNT at 100 mA g^−1^. SWCNTs are also electrochemically active materials and have shown upgraded performance in LIBs. As displayed in Figure S1, the cycle performance of SWCNT alone shows a capacity of 320 mA g^−1^. This demonstrates that SWCNTs contribute significantly to electrochemical performance due to their high surface region, good electrical conductivity, and ability to accommodate charge and discharge cycles efficiently. The steady cycling performance further features the capability of SWCNTs as excellent materials for improving the overall efficiency and lifespan of LIBs. Figure 4b,c displays the GCD measurement for PANI and PANI/SWCNT composite within the standard cycle range (0.01–3.0 V versus Li/Li^+^). These measurements assess the energy storage capacity and cycling stability of the anode materials in LIB. The GCD profile was recorded at various current densities and voltages using a battery testing system. The results indicate that the initial capacities of PANI and PANI/SWCNT anode electrodes were 760 and 1317 mA g^−1^, respectively. At 100 mA g^−1^, the initial coulombic efficiencies for PANI and PANI/SWCNT anode were 84.9 and 92.4% respectively. Figure 4d illustrates the rate capacity examination for the PANI, and PANI/SWCNT at various current densities of 0.1, 0.2, 0.5, 0.6, and 0.8 A g^−1^. The data show that electrode capacity decreases rapidly as current density increases. PANI electrode capacities of 559, 474, 402, 400, and 388 mA g^−1^ were observed at the respective current densities. The PANI/SWCNT electrode exhibited discharge capacities of 827, 682, 576, 575, and 561 mA g^−1^ at the same current densities. When the current density returned to its initial level (0.1 A g^−1^), the capacity was restored to 560 mA g^−1^ and 860 mA g^−1^. The capacity retention rates were 100.17% and 103.90%, showing improved charge and discharge capabilities. Table S2 provides detailed average capacities at various current densities for both electrodes.

This high capacity demonstrates that the composite is suitable for storing a greater amount of Li^+^, resulting in improved energy density. In addition, after 200 cycles, the composite held 101% of its initial capacity, exhibiting excellent cycling stability. This stability is possible because of the synergetic effect between PANI and 10 wt% SWCNTs, which improves the structural integrity of the composite and prevents specific-capacity degradation over repeated charge–discharge cycles.

**Figure 5:**
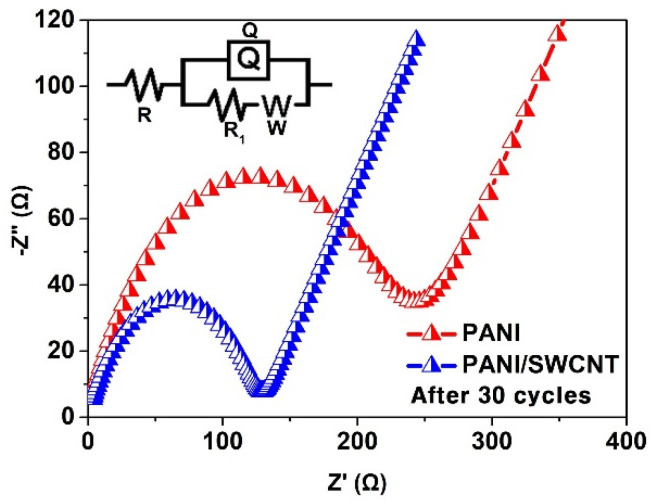
Nyquist plots from EIS for PANI and PANI/SWCNT after 30 cycles, along with the corresponding model for EIS fitting.

To better understand the superiority of PANI/SWCNT (10 wt%) over its parent PANI, EIS examination was performed. Figure 5 shows the Nyquist plots of PANI and PANi/SWCNT in a fully charged state (3.0 V vs. Li/Li^+^) for the 30 cycles. In the equivalent circuit (Figure 5, inset), R is the resistance of the solution, R_1_ is the electron transfer resistance, Q is the constant phase element, and W is the Warburg impedance. R_1_ is related to the charge transfer resistance extracted from the semicircle of the intermediate frequency; the Warburg impedance is indicative of Li^+^ diffusion dynamics at low frequencies. As summarized in Table S3, the R_1_ value for PANI is 238.3 and PANI/SWCNT is 128, which is lower than the PANI. The significantly reduced R_1_ for PANI/SWCNT synergistically reduces the volume expansion and increases the electrical conductivity.

Overall, the PANI/SWCNT composite exhibited superior electrochemical performance, displaying its potential as a high-specific capacity, stable, and conductive anode material for advanced LIBs. The upgraded redox activity, high-specific capacity, excellent cycling strength, and low impedance make it a promising candidate for advanced next-generation energy storage systems.

### 3.3. Li^+^ Diffusion Mechanism

The gradient structure of PANI on SWCNTs plays a significant role in improving Li^+^ diffusion, a critical consideration for the performance of LIBs. This construction synergistically combines the remarkable unique properties of PANI and SWCNTs to make an optimized pathway for efficient ion transport and storage. PANI, with its conductive polymer spine, works with reversible redox reactions, giving flexible and accessible destinations to Li^+^ intercalation. This upgrades the material’s ability to manage repeated charge-release cycles while maintaining high capacity and dependability. Then again, the SWCNTs contribute their high surface region, permeable nature, and remarkable conductivity to the PANI/SWCNT composite. SWCNTs ensure uniform electron and ion transport all through the material. The enormous surface region additionally advances a convincing association with the PANI coating, forestalling bottlenecks in the transport pathways. The intimate incorporation of PANI and SWCNTs inside this gradient structure limits diffusion resistance by ensuring reliable electron and ion advancement and developments. This tight coupling effectively reduces energy losses and amplifies Li^+^ storage capacities. Accordingly, the composite shows huge superior electrochemical performance, making it a great and excellent contender for advanced next-generation LIB anodes. The cooperative collaboration between PANI’s flexibility and redox movement with SWCNT’s conductivity and structural stability features the composite’s potential capacity as a predominant performance energy storage material.

The PANI faces several challenges for large-scale adoption. Scalability is a challenge due to the complex chemical processes involved in synthesis, which can be difficult to scale up while maintaining uniformity and reproducibility. The high cost of SWCNTs is a major drawback, impacting the composite’s cost-effectiveness. Long-term cycling stability is also a concern, as PANI is prone to structural degradation due to repeated volumetric expansion and contraction during charge–discharge cycles. Further material engineering, such as crosslinking, doping, or hybridization with other conductive materials, is needed to enhance durability. Uniform dispersion of SWCNTs within the PANI matrix is challenging due to strong van der Waals interactions, leading to inconsistencies in conductivity and electrochemical performance. Environmental factors, such as moisture and temperature variations, can affect the composite’s performance, necessitating encapsulation or protective coatings. Addressing these challenges through scalable synthesis methods, cost-effective alternatives for SWCNTs, and structural modifications to improve long-term stability is crucial for the practical implementation of PANI/SWCNT composites in energy storage devices. When contrasted with other polymer/carbon-based anodes, featuring its potential as a high-performance material for LIBs, the composite notably achieved an efficient high-specific capacity limit of 830 mA g^−1^ at 100 mA g^−1^, which is fundamentally higher than the other polymer/carbon-based anodes. For better understanding, the detailed compaction was tabulated in Table 1.

## 4. Conclusions

This study features the advancement of PANI and SWCNT composites as high-performance anode materials for LIBs. The composite exhibited a significant advancement, and improvements in specific capacity, cycling stability, and rate capacity, exhibiting its potential and promising alternative for advanced next-generation energy storage systems. The synergistic interaction between PANI and SWCNTs was viewed as the key component adding to the improved performance. PANI gave a flexible and conductive matrix that worked with reversible Li^+^ intercalation and de-intercalation, while SWCNTs offered a high-surface-area platform that ensured proficient electron transport and structural stability. Together, these elements feature minimized charge transfer resistance and supported fast Li^+^ diffusion, resulting in a specific capacity of 830 mA g^−1^ and outstanding specific capacity retention over 200 cycles. This work exhibits that incorporating conductive polymers like PANI with nanostructured carbon materials, such as SWCNTs, can open additional opportunities for improving upon the electrochemical performance of LIBs. The discoveries provide a pathway for developing advanced anode materials that can meet and fulfill the growing demands for effective, strong, durable, and high-energy-density energy storage capacity solutions. Future examinations may focus on increasing the synthesis process and further tuning the composite structure to boost performance for practical applications.

## Figures and Tables

**Table 1:** Electrochemical performance of various polymer/carbon-based materials as anode materials for LIBs.

Electrode	Specific Capacity (mAh g^−1^)	Currnet Density (mA g^−1^)	Cycles	Ref.
PDCDA-1100	290.9	200	100	[26]
C/Fe_3_C composite	750	100	120	[27]
SnO_2_/N-PC Fe_2_O_3_/N-PC	775 612	1000	200	[28]
Co_3_O_4_ nanoparticles	585.6	50	200	[29]
TNY-Pc	610	140	1150	[30]
CuTAPc	236	50	200	[31]
MA–VA–PcNi polymer	507	200	400	[32]
CoPc@CNF	740	1200	500	[33]
CoTAPc-PDA CoTAPc-BDA CoTAPc-TDA	602 701 865	100	300	[34]
YbPc-900 LaPc-1000	526.1 587.3	1000	300	[35]
CoPPc	514	200	550	[36]
rGO/FePc	186	300	100	[37]
MoS_2_/PANI	915	1000	200	[38]
SnO_2_/PANI	772	100	100	[39]
PANI PANI/SWCNT	528 830	100	200	This Work

## Data Availability

Date was created by own experiments and analysis and available on request.

## Supplementary Materials

The following supporting information can be downloaded at: https://www.mdpi.com/article/10.3390/polym17040478/s1. Figure S1. Cycle performance (at 100 mAh g^−1^) of SWCNT. Table S1. Performance comparison of *PANI* and *PANI/SWCNT* anode materials. Table S2. The comparison of the rate capacity of the *PANI* and *PANI/SWCNT* electrode. Table S3. Equivalent circuit parameters for the modified electrodes.
