# Supplementary material for: Design and Optimization of Polyaniline/SWCNT Anodes for Improved Lithium-Ion Storage
Source: Polymers (Basel). 2025 Feb 12;17(4):478. doi: 10.3390/polym17040478 (PMC11860079; doi:10.3390/polym17040478)
Supplement: Supplementary file 1 [file polymers-17-00478-s001.zip › polymers-3445848-supplementary.pdf]

Supplementary Information

Design and Optimization of Polyaniline/SWCNT Anodes for Improved Lithium-Ion Storage

Keshavananda Prabhu Channabasavana Hundi Puttaningaiah*

Department of Chemical, Biological, and Battery Engineering, Gachon University, Gyeonggi-do, Seongnam-si 13120, South Korea.

Email: keshavmgm@gmail.com

Correspondence should be addressed to Dr. Keshavananda Prabhu Channabasavana Hundi Puttaningaiah (keshavmgm@gmail.com)

Figure

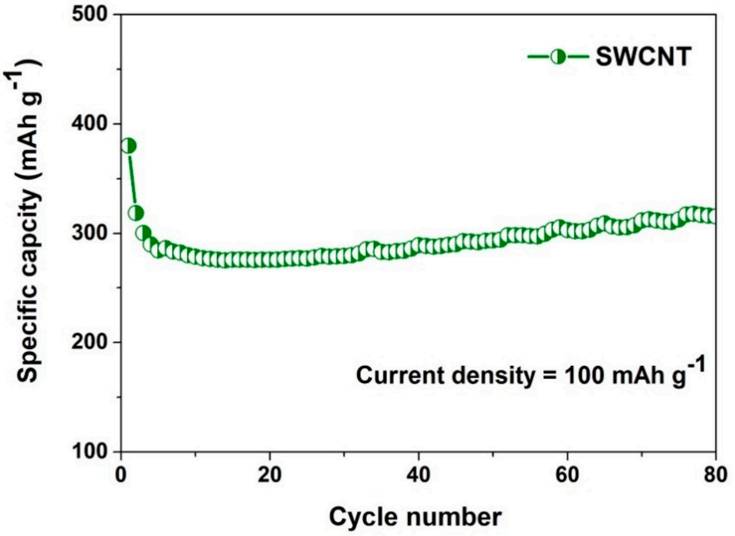

Figure S1. Cycle performance (at 100 mAh g^{-1}) of SWCNT.

Table

Table S1. *Performance comparison of PANI and PANI/SWCNT anode materials.*

Materials	Initial discharge capacity (mA g^{-1})	1st discharge capacity. (mA g^{-1})	Capacity retention after 100 cycles (%)
**PANI**	760	465	69
**PANI/SWCNT**	1317	836	63

Table S2. *The comparison of the rate capacity of the PANI and PANI/SWCNT electrode.*

Rate capacity [mAh g^{-1}]	0.1 A g^{-1}	0.2 A g^{-1}	0.5 A g^{-1}	0.6 A g^{-1}	0.8 A g^{-1}	0.1 A g^{-1}	Regained capacity (%)
**PANI**	559	474	402	400	388	560	100.17

**PANI/SWCNT**	827	682	576	575	561	860	103.90

Table. S3. Equivalent circuit parameters for the modified electrodes

**Electrode**	**R (Ω)**	**R_1 (Ω)**	**Q-Yo**	**Q-n**	**W (Ω)**
**PANI**	0.1	238.3	$9.38X10^{-6}$	0.8	0.002783
**PANI/SWCNT**	0.01	128	$2.74X10^{-5}$	0.6427	0.02954
